# Analyzing voter behavior on social media during the 2020 US presidential election campaign

**DOI:** 10.1007/s13278-022-00913-9

**Published:** 2022-07-18

**Authors:** Loris Belcastro, Francesco Branda, Riccardo Cantini, Fabrizio Marozzo, Domenico Talia, Paolo Trunfio

**Affiliations:** DIMES Department, University of Calabria, Rende, Italy

**Keywords:** Social media analysis, Opinion mining, User polarization, Sentiment analysis, Political events

## Abstract

Every day millions of people use social media platforms by generating a very large amount of opinion-rich data, which can be exploited to extract valuable information about human dynamics and behaviors. In this context, the present manuscript provides a precise view of the 2020 US presidential election by jointly applying topic discovery, opinion mining, and emotion analysis techniques on social media data. In particular, we exploited a clustering-based technique for extracting the main discussion topics and monitoring their weekly impact on social media conversation. Afterward, we leveraged a neural-based opinion mining technique for determining the political orientation of social media users by analyzing the posts they published. In this way, we were able to determine in the weeks preceding the Election Day which candidate or party public opinion is most in favor of. We also investigated the temporal dynamics of the online discussions, by studying how users’ publishing behavior is related to their political alignment. Finally, we combined sentiment analysis and text mining techniques to discover the relationship between the user polarity and sentiment expressed referring to the different candidates, thus modeling political support of social media users from an emotional viewpoint.

## Introduction

In recent years, the growing use of social media is generating an amount of information-rich data never seen before. This data, commonly referred as Big Social Data, can be effectively leveraged by a wide range of techniques aimed at modeling the interactions of users on social media, their collective sentiment and behavior, the dynamics of public opinion, and the patterns of information production (Pang and Lee 2008; Cesario et al. 2016; Marozzo and Bessi 2018; Cantini et al. 2022). All the knowledge extracted through such techniques allows to outline a precise profile of social users, by describing them from a behavioral and psychological viewpoint, and by modeling their perception of events and public decisions.

This manuscript presents an in-depth analysis of the posts published on Twitter during the 2020 US election campaign, aiming at outlining an accurate view of the political event from different points of view. Specifically, several techniques of topic discovery, opinion mining, and emotion analysis were combined in a unified data analysis workflow for investigating: (*i*) trending topics and their evolution over time, (*ii*) users’ political alignment and publishing behavior, and (*iii*) users’ sentiment and emotional aspects.

Firstly, we extracted the main discussion topics characterizing the 2020 US election campaign by leveraging the unsupervised approach proposed in Cantini et al. (2021), which relies on the density-based clustering of the latent representation of trending hashtags. Afterward, in order to achieve a more accurate representation of social media conversation, we studied the weekly evolution of the detected topics, which is useful to understand how online discussion evolves over time.

Secondly, we modeled the political alignment of social media users, in order to understand which candidate or party public opinion is most in favor of in the weeks preceding the Election Day. For this purpose, we exploited IOM-NN (*Iterative Opinion Mining using Neural Networks*), a neural-based opinion mining methodology we previously proposed in Belcastro et al. (2020). Specifically, a real-time analysis was carried out during the 2020 US presidential election campaign using data gathered from Twitter, correctly determining Joe Biden’s lead over Donald Trump before the Election Day. The achieved results, publicly available through our university web portal,^1^ represent a remarkable step forward with respect to previous works present in the literature. In fact, to the best of our knowledge, experimental evaluations are carried out after the end of the considered event, while in our case we have given a proof of the real-time effectiveness of IOM-NN, which leads to the possibility of using it for enhancing or even replacing traditional opinion polls. Furthermore, for the sake of completeness, we extended the results of the real-time analysis by focusing on the main swing states, i.e., those states characterized by a high uncertainty about the winning candidate and for this reason by a marked strategic importance. We assessed the statistical significance of the collected data by studying the age, gender and geographical distribution of Twitter users for understanding whether they can be considered voters of the political event. The obtained results confirm the great effectiveness of our approach, which outperformed the average of the latest opinion polls by correctly identifying the leading candidate before the Election Day in 10 out of 11 swing states. Furthermore, the polarization information achieved by IOM-NN was also leveraged to investigate the temporal dynamics of social media conversation, with the aim of studying how users’ publishing behavior is related to their political alignment, and how it reflected the occurrence of external events like debates or rallies.

Thirdly, we analyzed the relationship between the emotional sphere of Twitter users and their political alignment. In particular, we jointly exploited sentiment analysis and text mining techniques for extracting the sentiment of social media users. Then, we combined this information with the polarization achieved by IOM-NN for investigating how a user refers to the candidates while supporting his/her preferred faction, with respect to a broad spectrum of emotions. This step is useful for understanding how the supporters of a particular candidate express their preference on social media. Specifically, they can praise, as in the case of pro-Trump users, their favorite candidate with positive content that shows emotions like joy and confidence. Alternatively, they may be more likely to discredit the opposing candidate, as in the case of pro-Biden users, by producing negative online content characterized by emotions like anger, disgust and sadness.

The rest of the paper is organized as follows. Section 2 reports the most relevant approaches in computational politics and sentiment analysis present in the literature. Section 3 describes the different techniques combined in the proposed analysis workflow. Section 4 describes the experimental evaluation. Finally, Sect. 5 concludes the paper.

## Related work

Computational politics is a research area that involves a set of techniques aimed at analyzing users’ behavior during a political event of interest, both modeling and influencing their perception and opinion about facts, events and public decisions. With the rapid growth of social media usage, microblogging platforms have become a rich source of valuable information, which can be effectively exploited for investigating the patterns of information diffusion, the interactions between users and their opinion about a specific faction or candidate (Belcastro et al. 2020). According to a recent survey (Haq et al. 2020), existing literature on computational politics can be categorized into five classes, as discussed in the following.

*Community and user modeling* This class of works focuses on modeling the behavior of social media users from both an individual and collective viewpoint. Many works in this category are related to the analysis of homophily, i.e., the connection of groups of users driven by common interests, which leads to the formation of community structures of like-minded people (Grevet et al. 2014; Bastos et al. 2013; Fraisier et al. 2017). Other works focus on modeling political affiliation of social users, exploiting community information for predicting the results of a political event (Belcastro et al. 2020; Chiu and Hsu 2018; Takikawa and Nagayoshi 2017).

*Information flow* These works investigate how information flows within the network. Most of them analyze the misinformation spread, trying to detect fake news thus limiting its distortion effects on public opinion (Kim et al. 2018; Ciampaglia et al. 2015; Gyongyi et al. 2004). Other works in this category are also aimed at identifying echo chambers, i.e., situations in which the repetition and sharing of information causes the strengthening of an opinion inside a community (Garimella et al. 2018; An et al. 2013; Shu et al. 2019).

*Political discourse* Works in this category model online discussion from different points of view, taking into account demographic aspects, community structure and information diffusion patterns. Many works are aimed at extracting the main topics of discussion through topic modeling (Greene and Cross 2015; Trabelsi and Zaïane 2019), or identifying political crisis (Keneshloo et al. 2014). Opinion mining techniques can be also exploited for identifying the opinion or mood of social media users about those topics, as users’ interactions on social media can affect their political engagement (Hoffmann and Lutz 2017; Azarbonyad et al. 2017; Monti et al. 2013).

*Election campaigns* Research contributions in this class are aimed at measuring the engagement of the online audience, enabling large-scale opinion polls and the management of the political campaign. In fact, social media provide an effective platform for engaging users in political discussion, which is often used by politicians during the political campaigns (Wulf et al. 2013; Hong and Nadler 2015). Moreover, the analysis of political engagement of social users can accurately forecast the final results of the political event under analysis (Belcastro et al. 2020; Saleiro et al. 2016).

*System design* Works in this category propose a full system design of computation politics systems. As an example, Cambre et al. (2017) propose a system design that can help to break the echo chamber effect, moderating the online political discussion, while Dade-Robertson et al. (2012) discuss the relationship between political processes, urban environments and situated technologies.

In this work we use opinion mining and sentiment analysis techniques in order to investigate the polarization of the US social media users toward the different candidates involved in the 2020 US presidential election. Starting from this, we identify the emotional state (mood) of social users and its relation with their political orientation. Finally, we exploit the results of polarization analysis in order to forecast the final results.

There are several works in the literature that rely on text mining and natural language processing algorithms for investigating the opinion of social users and their collective sentiment toward political candidates or parties. Oikonomou and Tjortjis (2018) used Textblob,^2^ a Python library for natural language processing, to predict the outcome of the US presidential election in three states of interest (i.e., Florida, Ohio and North Carolina). Wong et al. exploited (Wong et al. 2016) SentiStrength,^3^ a lexicon-based sentiment analysis tool, for modeling the political behaviors of users by analyzing tweets and retweets. Alashri et al. (2016) analyzed Facebook posts about the 2016 US presidential election with CoreNLP^4^ (Manning et al. 2014), one of the most popular tool for natural language processing, to examine the dynamics between candidate posts and comments they received on Facebook and calculate a score for each political candidate for measuring his/her credibility on a given issue. Singh et al. (2021) carried out a comparison among four machine and deep learning algorithms (i.e., TextBlob, Naive Bayes, SVM, and BERT (Devlin et al. 2018)) for sentiment analysis. Authors used the 2020 US presidential election as a case study, finding that the use of BERT leads to the best results.

All of the aforementioned techniques are characterized by several issues related to the use of social media data for predicting the outcome of political events, which are *language barrier*, *misclassification*, *data imbalance* and *reliability* (Bilal et al. 2019). Consequently, in order to achieve a precise estimate of the political polarization of the US citizens, we leveraged the IOM-NN technique, specially designed to overcome these issues (Belcastro et al. 2020): (*i*) it is language-independent, as it uses a hashtag-based bag of words representation; (*ii*) it avoids misclassifications using a high threshold on the polarization probability; (*iii*) it uses randomized class balancing algorithms in order to avoid the learning process being biased toward majority classes; (*iv*) it requires a preliminary study of users’ representativeness, in order to understand whether they can be considered voters in the political event under analysis (See Sect. 4.1.1). Moreover, with respect to state-of-art techniques, IOM-NN allows the classification of a much greater number of tweets and users, due to its incremental and iterative nature, which leads to a better quality and robustness of the results.

## Analysis workflow

In this work we present an in-depth analysis of the posts published on Twitter during the 2020 US election campaign, with the aim of outlining an accurate representation of this political event from different perspectives, including users’ publishing behavior, discussion topics, political alignment and its relationships with the emotional sphere.

**Fig. 1:**
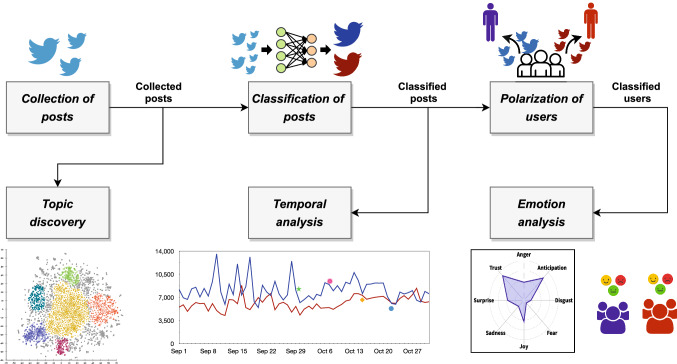
A graphic representation of our analysis workflow

For this purpose, several techniques were combined in a unified analysis workflow, represented in Fig. 1, composed of the following steps:

- *Collection of posts*: data are gathered from social media by using a set of keywords related to the considered political event.
- *Classification of posts*: the collected posts are classified in favor of a faction according to the detected political support.
- *Polarization of users*: the classified posts are analyzed for determining the polarization of users toward a faction.
- *Topic discovery*: the collected posts are analyzed in order to identify the politically related discussion topics underlying the conversation on social media, modeling their evolution over time.
- *Temporal analysis*: the temporal dynamics of social media conversation are analyzed and combined with the polarity information of classified posts in order to study users’ publishing behavior in relation to their political alignment.
- *Emotion analysis*: the polarized posts are exploited for investigating the relationship between the political orientation of users and the different emotions they expressed in referring to the different candidates.

Among the aforementioned steps, the first three jointly constitute the IOM-NN methodology (Belcastro et al. 2020), while the fourth follows the approach to topic detection in social data proposed in Cantini et al. (2021). IOM-NN is an opinion mining technique aimed at discovering the political polarization of social media users during election campaigns characterized by the competition of political factions. The methodology relies on an iterative and incremental procedure based on feed-forward neural networks, aimed at discovering the political polarization of social media users by analyzing the posts they publish. An open-source implementation of IOM-NN is available on Github.^5^

In the following sections we provide a detailed description of the proposed analysis workflow. Moreover, to facilitate understanding of the different steps, we will show practical examples by examining a small subset of the collected data.

### Collection of posts

The goal of this step is to collect a set $\mathcal {P}$ of social media posts from different sources (e.g., Twitter), related to the political event $\mathcal {E}$ under analysis. As a first step, the different factions, parties or candidates involved in the political event are identified, defined as the set $\mathcal {F} = \{f_1, f_2, \dots , f_n\}$. In particular, in the case of the 2020 US presidential election, we focused on the two main candidates Joe Biden and Donald Trump. Afterward, geotagged posts are gathered by using a set of keywords $\mathcal {K}$ that is partitioned as follows:

- *neutral keywords* ($\mathcal {K}_{context}$) that contains generic keywords that can be associated to $\mathcal {E}$ without referring to any specific faction in $\mathcal {F}$(e.g., $\#vote$, $\#election2020$);
- *faction keywords* ($\mathcal {K}^\oplus _{\mathcal {F}} = {\mathcal {K}^\oplus _{f_1}, \dots , \mathcal {K}^\oplus _{f_n}}$) that contains the keywords used for supporting each faction (e.g., $\#votebiden$, $\#maga$).

The keywords selection process requires a small amount of domain knowledge, as these keywords can be manually selected among the trending hashtags that people commonly use to refer to $\mathcal {E}$ on social media. Moreover, the keyword selection process can be automatized by searching for specific patterns, like “#vote + *candidate*”, often used for labeling politically polarized posts. We assessed the statistical significance of the collected posts by studying the age, gender and geographical distribution of Twitter users for understanding whether they can be considered voters of the political event. For this purpose we used a wide range of information which can be directly extracted from users metadata (e.g., location and language), or examined starting from statistical reports about the usage of the social media platform in a given country (e.g., user distribution by age and gender). Furthermore, in order to improve the representativeness of the collected posts, user accounts are analyzed, filtering those that show anomalous publishing activity, such as social bots or news sites, or those that have inconsistent information in their profile, such as for example a location that is not defined or does not belong to any of the states considered (see Sect. 4.1.1).

**Fig. 2:**
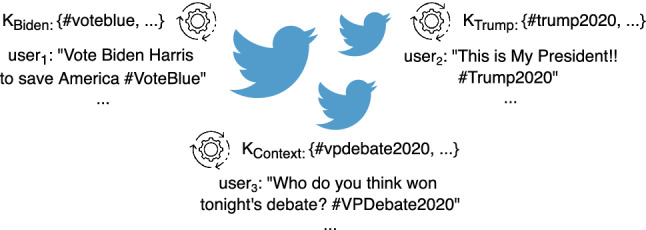
Example of how the *collection of posts* step works

Collected posts undergo the following preprocessing operations: *i*) the text of each post is converted to lowercase and accented characters are normalized; *ii*) words are lemmatized and stemmed (e.g., vote or votes or voted $\xrightarrow {\text {}}$ vot); *iii*) stopwords are removed; and *iv*) bigrams are identified (e.g., San Francisco $\xrightarrow {\text {}}$ San$\_$Francisco). Figure 2 shows an example of how posts are collected using keywords about the 2020 US presidential election. Some of these keywords are generic (e.g., $\#vpdebate2020$), and others are used to support a specific candidate (e.g., $\#voteblue$ for Biden and $\#trump2020$ for Trump).

### Classification of posts and temporal analysis

**Fig. 3:**
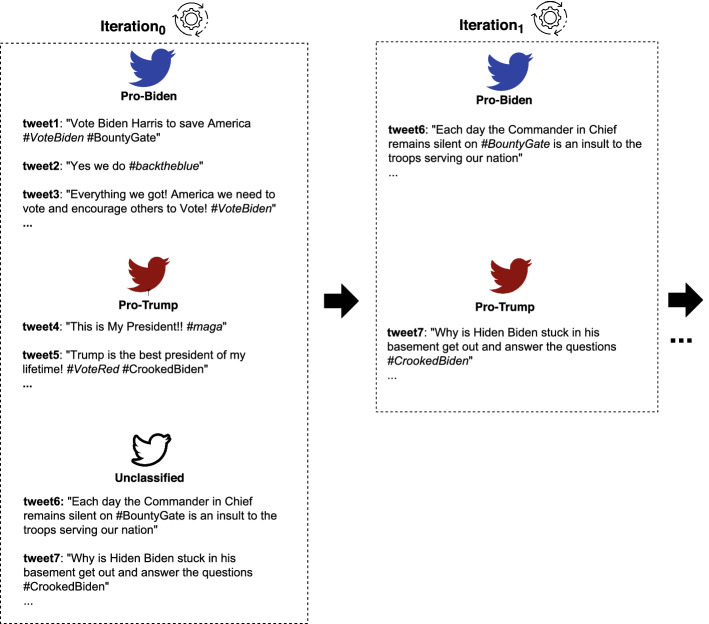
Example of how the *classification of posts* step works

During this step, the posts $\mathcal {P}$ collected in the previous step are classified in favor of a faction by using IOM-NN. Specifically, a preliminary iteration is performed for classifying input posts according to the keywords in $\mathcal {K}^\oplus _{\mathcal {F}}$. Posts containing keywords related to exactly one faction are polarized toward that faction, while remaining posts are labeled as neutral. Then, neutral posts undergo an iterative classification process, during which the model exploits the knowledge acquired at the previous iterations. It is worth noticing that, due to the incremental nature of the annotation process, IOM-NN is not tied to a specific set of initial faction keywords and does not require an in-depth knowledge of the political event under consideration. In fact, even starting from a small but representative set of faction keywords, IOM-NN is able to infer new classification rules iteratively, which implies a good robustness and generalizability of the methodology. Figure 3 shows a classification example of a small set of tweets about the 2020 US presidential election, which exploits the following faction keywords.

- $$\mathcal {K}^\oplus _{Biden}$$ = $$\{\#voteblue$$, $$\#backtheblue$$, $$\#votebiden$$, ...$$\}$$;
- $$\mathcal {K}^\oplus _{Trump}$$ = $$\{\#votered$$, $$\#trump2020$$, $$\#maga$$, ...$$\}$$.

At iteration 0, IOM-NN uses the keywords in $\mathcal {K}^\oplus _{\mathcal {F}}$ for classifying five tweets. In the subsequent iterations, the neural model iteratively exploits the tweets classified in the previous steps for generating new classification rules based on co-hashtag relationships. As an example, at iteration 1 the model is trained with the tweet classified at iteration 0, discovering new political-oriented topics of discussion and generating the following classification rules:

- tweets with keywords $\#bountygate$ are classified in favor of Biden since Donald Trump was accused of paying Moscow’s secret agents for the killing of the US servicemen in Afghanistan;
- tweets with keywords $\#crookedbiden$ are classified in favor of Trump since Hunter Biden (i.e., second son of US President Joe Biden), was accused by Donald Trump of wrongdoing in regard to China and Ukraine.

This learning process iterates until the algorithm is no longer able to generate new classification rules and therefore to identify the polarization of new tweets.

After having classified the posts according to the polarity discovered by IOM-NN, this information is used for investigating how the user publishing behavior is related to their political alignment. Specifically, temporal dynamics of social media conversation are analyzed, studying how information is produced by the supporters of both candidates, and how this reflects the occurrence of external events such as debates and rallies.

### Polarization of users

**Fig. 4:**
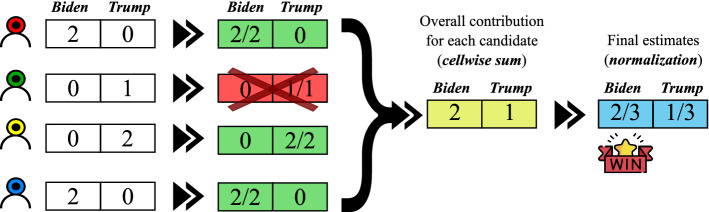
Example of how the *polarization of users* step works

This step is aimed at analyzing the set of previously classified posts in order to determine the *polarization of users* toward a faction. Specifically, the list of classified posts for each user *u* is computed, filtering out those users that published a number of posts below a given threshold. Afterward, a score vector $v^u_s$ for each user *u* is computed, which contains his/her score for each faction. Finally, IOM-NN calculates the overall faction score as the normalized sum of the score vectors. Figure 4 shows how the *polarization of users* step works on the classified posts reported in Fig. 3. For each user, the posts in favor of Biden and Trump are counted, discarding those users who have published less than two tweets. Then, the polarization vector for each user is computed containing the percentage of posts published in favor of his/her preferred faction. Lastly, the final score vector is determined, that contains the overall polarization percentages for the two candidates.

### Emotion analysis

This step analyzes the polarized posts for identifying the users’ sentiment underlying the online discussion about the presidential candidates. Specifically, we combined the information about the political alignment of social media users with their sentiment and emotional expressions. Firstly, in order to extract the sentiment from online published content, we exploited SentiStrength (Thelwall 2017) for the annotation of social media posts. In particular, for each polarized post we computed a positive $\mathcal {S}c^+(p)$ and negative $\mathcal {S}c^-(p)$ sentiment score, both ranging between 1 (neutral) and 5 (strongly positive/negative). Then, the overall sentiment score $\mathcal {S}c(p)$ of a polarized post is obtained as follows: $\mathcal {S}c(p) = \mathcal {S}c^+(p) - \mathcal {S}c^-(p)$. Secondly, we modeled the political orientation of social media users from an emotional point of view by exploiting NRC-EmoLex (Mohammad and Turney 2013), a publicly available emotion lexicon which has proven its performance in several sentiment and emotion classification tasks, as described in  Kiritchenko et al. (2014),  Mohammad (2012), and  Nakov et al. (2016). Specifically, NRC contains more than 14 thousand English terms labeled by the expressed polarity (i.e., positive or negative) and eight basic emotion categories of Plutchik (2001) (i.e., joy, trust, anticipation, sadness, surprise, disgust, fear or anger). Finally, we combined the obtained information with the political alignment discovered by using IOM-NN, in order to extract the overall sentiment and emotions expressed by social media users, while talking about the two candidates.

**Fig. 5:**
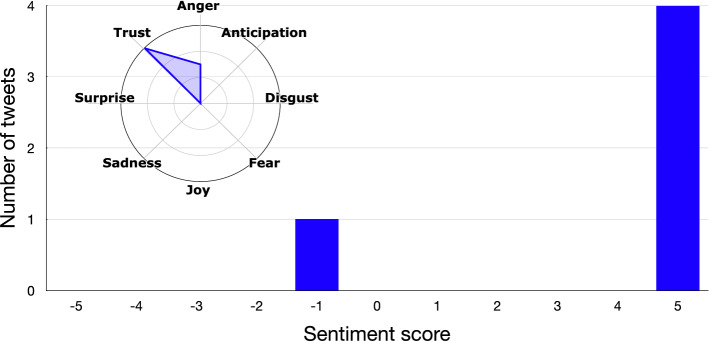
Example of pro-Biden tweets

**Fig. 6:**
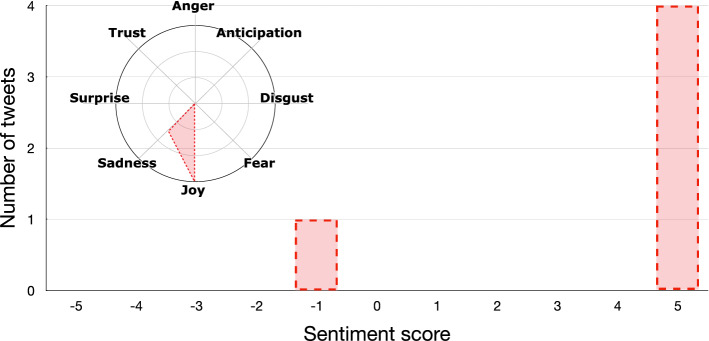
Example of pro-Trump tweets

As an example, Figs. 5 and 6 show how the *sentiment analysis* step works on the polarized tweets obtained at the previous step (i.e., a small subset of the collected tweets) for understanding the emotional state of the users who support the different candidates. As we can see, polarized tweets are quite positive for both candidates, but show different emotional profiles. In particular, Biden’s supporters show *trust* in the new presidential candidate, while Trump’s ones express their *joy* at having Trump as president.

### Topic discovery

This step is aimed at identifying the main politically-related discussion topics characterizing the 2020 US election campaign, by following the unsupervised approach used in Cantini et al. (2021). As a first step, a Word2Vec model is trained on the entire corpus of tweets, in order to get the latent representation of hashtags and words in a 150-dimensional vector space. We selected the dimension of the embedding space by conducting several experiments, finding out the smallest size for which a clear clustering structure emerged, i.e., the best trade-off between complexity and representativeness. Subsequently, all hashtags are embedded in that 150-dimensional space, whose dimensionality is then reduced by using the t-distributed stochastic neighbor embedding (t-SNE) technique, initialized through principal component analysis (PCA), to obtain a 2D projection of that space. Moreover, in order to reduce noise, all hashtags with a frequency lower than a given threshold are filtered out. Finally, the OPTICS algorithm is used for extracting a clustering structure based on the topic-based separation of hashtags, induced by the projection of their semantic distribution. We have chosen this clustering algorithm due to its ability to discover clusters with arbitrary shape. In addition, compared to classical density-based algorithms such as DBSCAN, it is able to extract clustering structures at different density levels, which in our work is useful for dealing with micro-topics.

## The US 2020 Presidential election analysis and experimental results

In this section we provide an accurate description of the results coming from the analysis of the 2020 US presidential campaign, characterized by a strong rivalry between Joe Biden and Donald Trump. In particular, we analyzed election-related tweets with the aim of outlining a precise representation of this political event from different points of view, in terms of users’ publishing behavior, sentiment, political alignment and discussion topics. For this purpose we combined several techniques in an analysis workflow, whose steps are accurately described in Sect. 3 and whose results are reported in the following sections.

### Data description

**Fig. 7:**
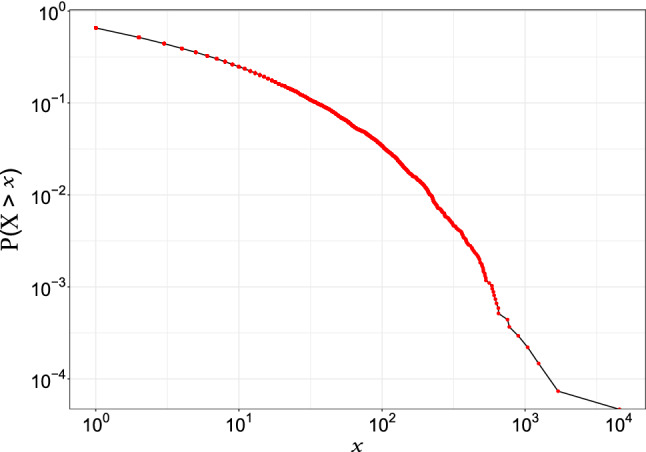
Complementary Cumulative Density Function (CCDF) of published tweets per user

The data used to perform the experimental evaluation comes from a public repository that contains a real-time collection of tweets related to the 2020 US presidential election from December 2019 to June 2021 (Chen et al. 2021). From such repository we considered only the tweets published close to the election event (from September 1 to October 31, 2020), i.e., about 160 million of which 18 million are tweets (11%), 110 million are retweets (69%), and 32 million are replies (20%), posted by about 29 million users. Only 22% of filtered data contain hashtags (e.g., $\#trump2020$, *#bidenharris2020*), useful to understand the arguments used in favor of the different candidates. In particular, the percentage of tweets published with at least one hashtag related to Trump (i.e., $\#trump$, $\#trump2020$, and $\#maga$) and Biden (i.e., $\#bidenharris2020$, $\#biden$) is about 31% and 11%, respectively. However, 7% of tweets contain at least one negative hashtag about Trump (i.e., $\#trumpknew$, $\#pedotrump$, $\#trumphascovid$, $\#trumptaxreturns$, *#bountygate*), whereas only 1% of tweets contain a negative hashtag for Biden (i.e., $\#crookedjoebiden$). In order to ensure the representativeness of the collected posts, we analyzed users’ account information, filtering out content posted by users that show an anomalous publishing activity or inconsistent profile information. This step allows to avoid the negative effects caused by the presence of content published by new sites and social bots, which can introduce a heavy bias in social media data (Cantini et al. 2022). We further analyzed the publishing behavior of the users in the filtered dataset by determining the Complementary Cumulative Density Function (CCDF) of shared tweets per user. Specifically, given the random variable *X* representing the number of shared tweets, it is determined by the frequency of users publishing a number of posts greater than *x*, i.e., the probability $P(X > x)$. The scatter plot in log-scale shown in Fig. 7, reveals a highly skewed distribution, with few active Twitter users posting a huge amount of tweets, and many users posting infrequently or not at all, the so-called social lurkers.

#### Statistical significance of collected data

Here we investigate the statistical significance of the collected data in order to assess users representativeness, i.e., whether they can be considered voters of the political event under analysis.

**Fig. 8:**
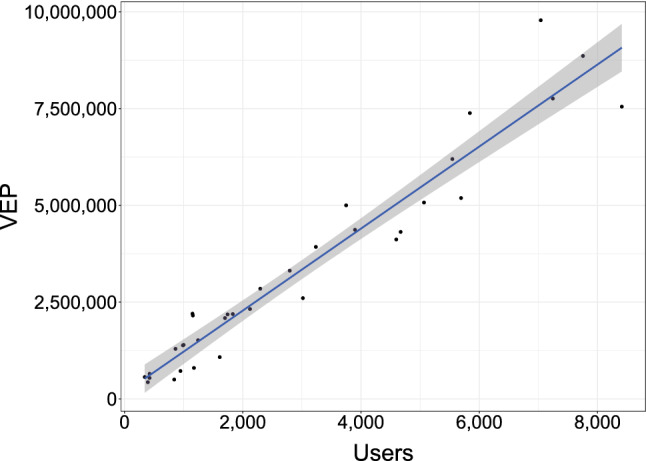
Linear interpolation: analyzed users versus voting-eligible population grouped by the US states

Firstly, from tweets metadata we extracted aggregate information on the used language of social media users, discovering that most of tweets have the *lang* field set to *English* (about 90%), whereas the remaining 10% is *Undefined* or set to other languages like *Spanish*. Secondly, we compared the number of Twitter users in our dataset, grouped by state, with the number of adult citizens actually living in that state, belonging to the voting-eligible population (VEP).^6^ Specifically, users were associated with states via Twitter metadata, by analyzing the *location* field present in each tweet, which indicates the location defined by the user in his/her Twitter account (e.g., Austin, TX). It is worth noting that, from the textual analysis of this field, it is not always easy to extract a meaningful city/state, as many users either left the field blank, or did not provide precise information (e.g., “USA”), or specified fictitious or nonexistent locations (e.g., “the moon” or “NY, Italy”). We measured the strength of this correlation, finding a Pearson coefficient $r=0.97$, significant at $p < 0.01$. The linear relationship that links users and the voting-eligible population can be easily seen in Fig. 8, which depicts an interpolation of the related scatter plot, with a goodness-of-fit $R^2=0.93$. Notice that outlier states were not considered in this step in order to achieve meaningful results, by excluding data of different magnitude. In addition, we explored age and gender distribution of analyzed users, finding out that about 94% of them are adults (at least 18 years old)^7^ and almost equally divided by gender .^8^

Among all the available tweets we have selected those published by users located in the 11 main swing states (i.e., *Arizona*, *Florida*, *Georgia*, *Michigan*, *Minnesota*, *Nevada*, *New Hampshire*, *North Carolina*, *Pennsylvania*, *Texas*, *Wisconsin*). We analyzed only these states as they are characterized by a marked political uncertainty and their outcomes have a high probability of being a decisive factor of the electoral event. We made this data, used in all the subsequent analysis steps, publicly available on Github.^9^

Table 1 reports a comparison between the users we were able to capture for each swing state and the VEP. The high correlation between the number of analyzed users per state and the VEP leads to a significant set of social media data effectively exploitable to determine the polarization of public opinion. However, despite the representativeness of the considered posts, the results achieved by the analysis of the online conversation can be influenced by platform biases. Specifically there exist usage biases due to the distribution of users of a social media platform in terms of gender, age, culture and social status, as well as technical biases related to platform policies about data availability and restrictions imposed in some areas of the world.

**Table 1:** Number of Twitter users versus voting-eligible population (VEP) grouped by swing states

State	#Users	#VEP
Arizona	5,692	5,189,000
Florida	16,921	15,551,739
Georgia	5,841	7,383,562
Michigan	8,411	7,550,147
Minnesota	4,596	4,118,462
Nevada	1,156	2,153,915
New Hampshire	1,610	1,079,434
North Carolina	7,245	7,759,051
Pennsylvania	7,040	9,781,976
Texas	19,119	18,784,280
Wisconsin	3,898	4,368,530

### Trending topics of the election campaign

**Fig. 9:**
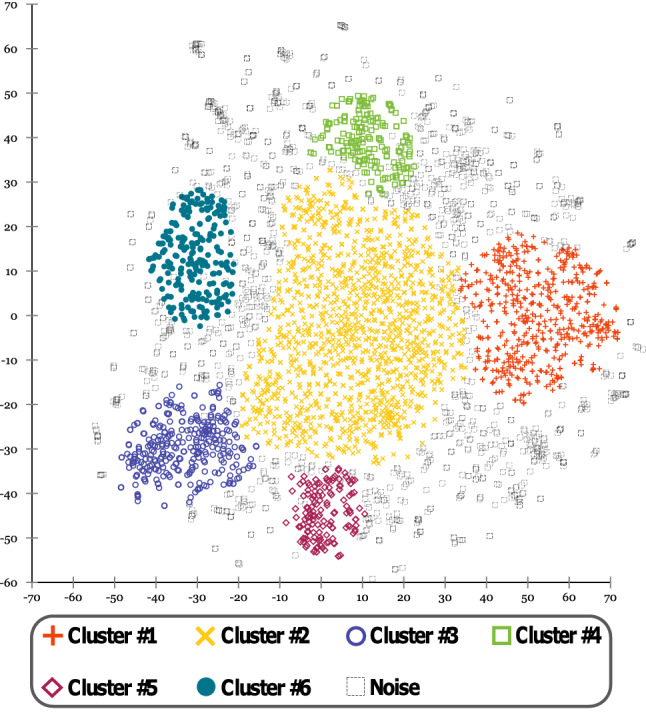
Unsupervised detection of the main topics underlying the online discussion

In this step we identified the main politically related discussion topics characterizing the 2020 US election campaign. Achieved results are shown in Fig. 9, where six clusters are clearly visible, each one related to a different topic of discussion. Moreover, Table 2 summarizes the discovered topics by reporting the corresponding top hashtags.

**Table 2:** Brief description of the identified topics

Cluster ID	Topic	Top hashtags
#1	Bad management of Covid-19 emergency	#trumpknew, #trumpvirus, #covid, #trumpisaloser, #trumpisanationaldisgrace, #trumpliedpeopledied
#2	Town hall meetings; sub-topics: climate crisis, veterans, discrimination	#cnntownhall, #climatecrisis, #greennewdeal, #respectveterans, #hererightmatters, #stoptrumpsterror
#3	Encouraging peopleto vote	#election2020, #voteearly, #vote2020, #votebymail, #voteready, #electionday
#4	Accusations against Hunter Biden	#hunterbiden, #bidencrimefamily, #burisma, #ukraine, #hunterbidenemails, #china
#5	The US Supreme Court;nomination of Amy Coney Barrett	#scotus, #amyconeybarrett, #filltheseat, #supremecourt, #riprbg, #scotushearings
#6	Support for Trump	#maga, #votetrump2020, #maga2020, #kag, #voteredtosaveamerica2020, #trumppence2020

The first topic is focused on the criticisms leveled at Trump regarding the management of the health emergency in the USA caused by Covid-19 pandemic. The second one is related to the online discussion about town hall meetings, covering different topics against Trump like discrimination, veterans and climate crisis (e.g., he referred to climate change as a “*hoax*”, and to veterans as “*human scum*”). The third one is a general topic about the presidential election. The fourth topic is related to the accusations of corruption and wrongdoing in regards to China and Ukraine leveled against Hunter Biden, i.e., the son of the democratic candidate Joe Biden. The fifth topic focuses on the nomination of the conservative Amy Coney Barrett for a seat on the Supreme Court as successor to the liberal Associate Justice Ruth Bader Ginsburg. Finally, the last topic is related to the online discussion of Trump’s supporters, characterized by notorious hashtags like *#maga* or *#kag*.

**Fig. 10:**
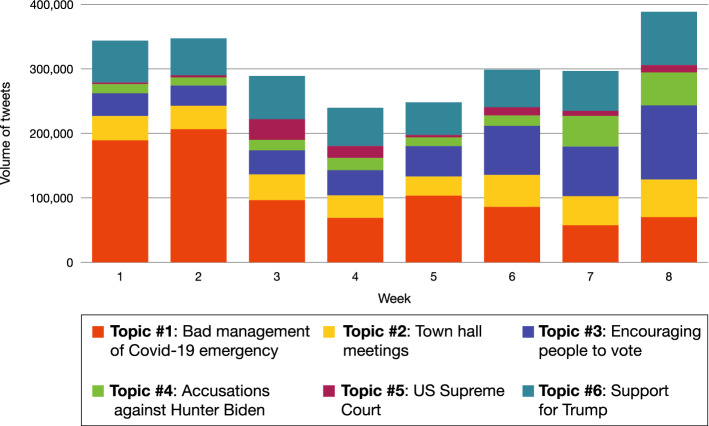
Weekly volume of tweets related to the detected topics from September 1 to October 31, 2020

Once the major discussion topics were detected, we analyzed their overall impact on the online conversation, along with their evolution in the eight weeks included in our observation period, as shown in Fig. 10. In particular, we calculated the volume of each hashtag-based topic by determining the percentage of tweets that contain hashtags belonging to the corresponding cluster. Considering our overall observation period, the most relevant topic is about Covid-19 pandemic and it specifically refers to Trump’s mismanagement of the health emergency. Other topics are related to the presidential election in general or arise from the publishing activity of Trump’s supporters. Also Biden’s supporters significantly contributed to the online discussion, by leveraging anti-Trump sub-topics that have emerged from several town hall meetings, about discrimination, veterans and the position of the Republican candidate about the climate crisis.

For what concerns the temporal evolution of the detected topics, we found that in the early weeks online conversation focused on the relationship between Trump and Covid-19 pandemic. In addition, the discussion about the US Supreme Court showed a slight increase close to the nomination, announced by Donald Trump, of Judge Amy Coney Barrett as Associate Justice of the US Supreme Court to fill the vacancy left by the death of Ruth Bader Ginsburg. In the following weeks, the focus of the online conversation shifted to various topics related to the approach of the Election Day and the importance of voting. We also observed an increase in the volume of tweets concerning the accusations leveled against Joe Biden’s son (i.e., Hunter Biden), a topic discussed mostly by the Democratic candidate’s detractors. Finally, other topics regarding the support voters expressed toward Trump and their criticisms leveled against him linked to town hall meetings showed an almost constant impact on the online conversation.

### Temporal analysis

In this step we investigated the temporal dynamics of social media conversation, in order to analyze users’ publishing behavior, studying how it is related to the detected polarity and how it reflected the occurrence of external events (e.g., debates, rallies, etc.). However, as described by the repository owners in Chen et al. (2021), there may be gaps in the dataset due to several issues. Firstly, the data collection step was highly contingent upon the stability of the network and hardware. Secondly, Twitter significantly limits the number of tweets that can be rehydrated. Finally, tweets may no longer be available as users have been removed, banned, or suspended.

**Fig. 11:**
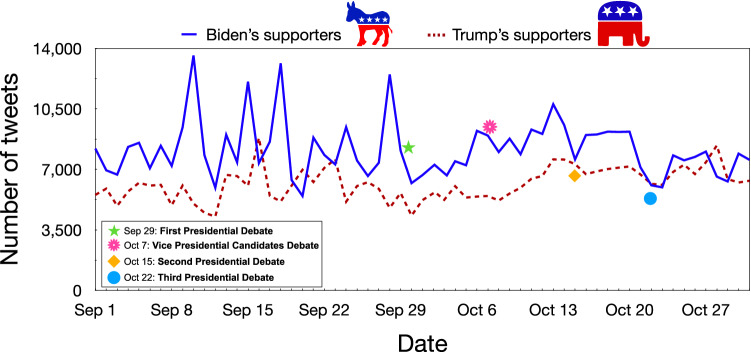
Time series of polarized tweets published from September 1 to October 31, 2020

Figure 11 shows the timeline of polarized tweets volume annotated with the four main political debates occurring during the election campaign, i.e., between September 1 and October 31. The first observation period (September 1 to September 28) exhibits significantly different communication dynamics prior to the first debate. Interestingly, this image shows an intense activity spikes of Biden’s supporters, as a likely consequence of President Trump’s actions:

- *September 10*: president Trump has attacked Democratic Vice Presidential candidate Kamala Harris.
- *September 15*: despite being banned by state authorities from holding rallies, President Trump still decided to hold one in Nevada.
- *September 18*: president Trump blamed blue states for the high number of the US Covid-19 fatalities.
- *September 28*: during a rally in Pennsylvania, Trump called Biden “a dishonest politician and a puppet in the hands of the radical left”.

The second and third observation windows (from September 30 to October 31) show typical weekly cycles of social media chatter, with no particular explosion or shock-related spike from external events, except for October 6 (before the Vice Presidential debate) and October 13 (before the second Presidential debate).

### Comparative analysis with opinion polls

In this step we assessed the effectiveness of our approach in determining the polarity of social media users with the aim of understanding which candidate or party public opinion is most in favor of. A first remarkable result was obtained through a real-time analysis, carried out on Twitter data collected during the two weeks before the Election Day. Specifically, IOM-NN was able to correctly determine Joe Biden’s lead over Donald Trump, especially in Georgia, where a Democratic candidate had not won since 1992 with the election of Bill Clinton. This promising result, publicly available through our university web portal,^10^ represents a step forward with respect to our previous work, as it gives a clear proof of the real-time effectiveness of IOM-NN, which suggests the possibility of using it to enhance or even replace traditional opinion polls.

Starting from the encouraging real-time results, we extended that analysis by focusing on the main eleven swing states, as described in Sect. 4.1.1. Specifically, we compared the results obtained through IOM-NN with the average values of the latest opinion polls before the election.^11^ For each analyzed state, Table 3 reports the real voting percentages, opinion polls, and IOM-NN estimates. The two candidates (i.e., Joe Biden and Donald Trump) are indicated with “*B*” and “*T*”, respectively. The winning candidate is written in bold when it is correctly identified.

**Table 3:** Comparison between voting percentages estimated by IOM-NN and the latest opinion polls

State	Real percentages		Opinion polls		IOM-NN	
	B	T	B	T	B	T
Arizona	49.4	49.1	**48**.**0**	45.8	**50**.**2**	48.3
Florida	47.9	51.2	48.7	46.0	48.0	**51**.**1**
Georgia	49.5	49.2	**47**.**6**	47.4	**52**.**7**	46.0
Michigan	50.6	47.8	**49**.**9**	44.4	**55**.**4**	43.0
Minnesota	52.4	45.3	**51**.**6**	41.8	**55**.**1**	42.6
Nevada	50.1	47.7	**49**.**4**	44.4	**49**.**8**	48.0
New Hampshire	52.7	45.4	**53**.**4**	42.4	**50**.**9**	47.3
North Carolina	48.6	49.9	47.8	47.5	56.6	41.9
Pennsylvania	50.0	48.8	**49**.**4**	45.7	**55**.**7**	43.1
Texas	46.5	52.1	47.5	**48**.**8**	46.1	**52**.**5**
Wisconsin	49.4	48.8	**52**.**0**	42.8	**56**.**3**	41.9
Correctly classified	–		**9/11**		**10/11**	
Tweets	–		–		670,451	
Users	–		$\approx$ 11,000		57,116	
Avg. Acc	–		0.82		0.91	

**Fig. 12:**
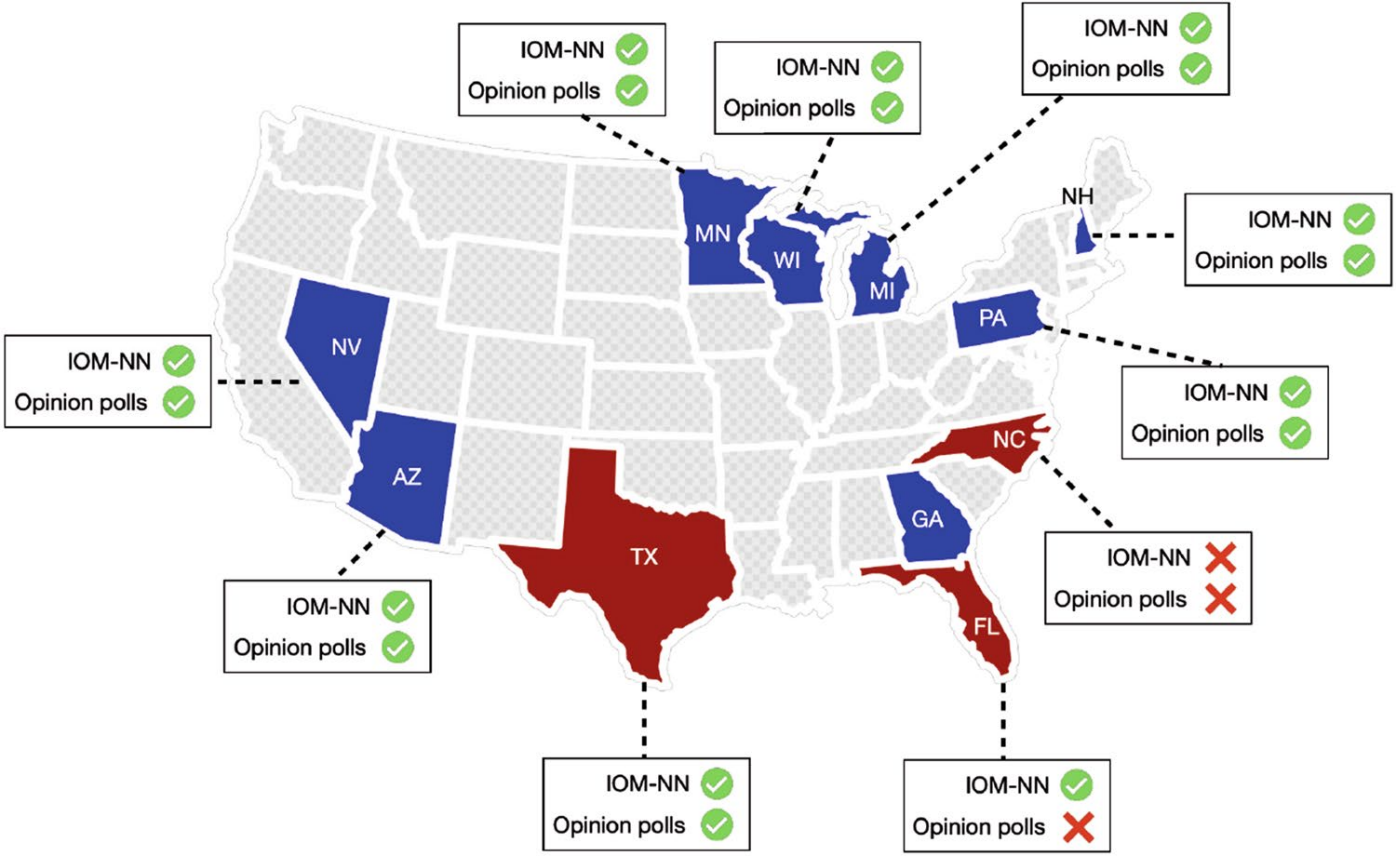
Comparison between IOM-NN and the latest opinion polls in identifying the winning candidate

The results of the comparison are summarized in Fig. 12, which shows that the estimates achieved by IOM-NN, related to the voting intentions of social media users are more in-line with the actual behaviors of voters with respect to the opinion polls, thus giving a clue to the final result in 10 out of 11 swing states (with an average accuracy of 91%). Using this metric we penalize the inversions of polarity which can be a crucial issue while analyzing these kinds of states characterized by a high degree of uncertainty. Notice that, for what concerns North Carolina, neither the estimates achieved by IOM-NN nor the opinion polls were in-line with the actual outcome in this state. This is a common situation as the results achieved by the polls and IOM-NN must be understood as an estimate of the polarization of public opinion in the weeks preceding the Election Day, not always in accordance with the actual behavior of voters. Moreover, a noteworthy advantage of IOM-NN with respect to traditional opinion polls, is the ability to capture the opinion of a larger number of people more quickly and at a lower cost. This makes IOM-NN a valid support to enhance or even replace opinion polls, by providing relevant insights useful to understand the dynamics of the election campaign.

### Emotion analysis

**Fig. 13:**
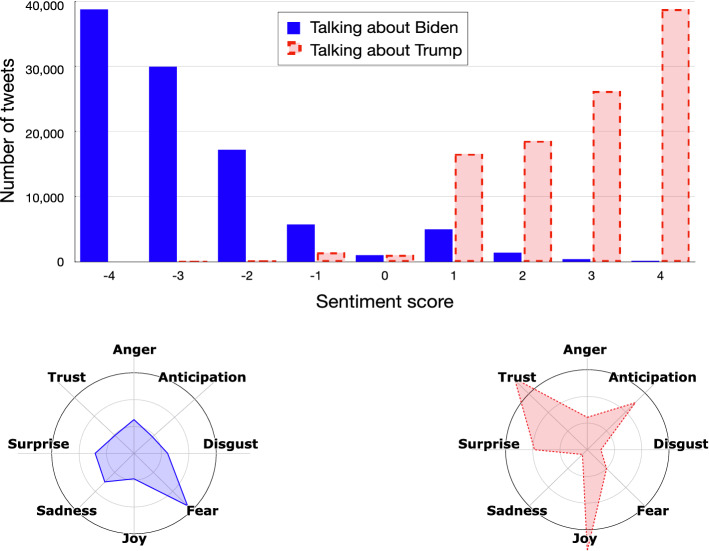
Distribution of sentiments and emotions of pro-Trump tweets

**Fig. 14:**
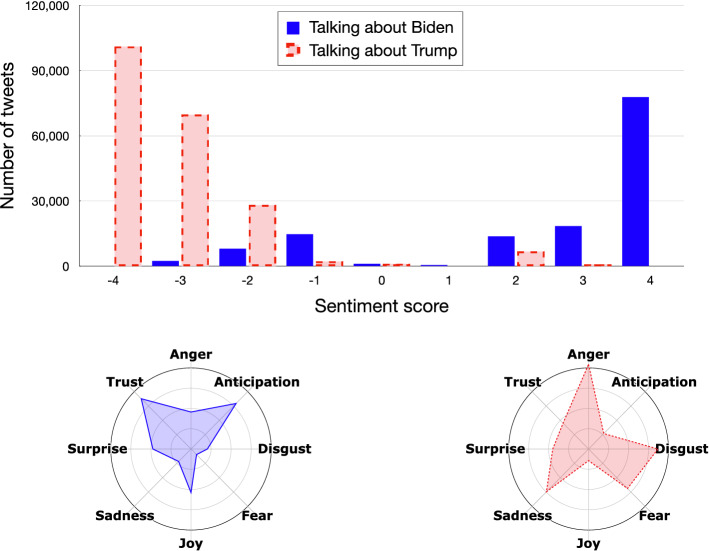
Distribution of sentiments and emotions of pro-Biden tweets

The goal of this last step is to model the political orientation of Twitter users from an emotional point of view. To this purpose, we used the SentiStrength tool (as explained earlier in Sect. 3.4), for discovering the existing relationships between user polarity and the sentiment expressed in referring to the two presidential candidates. Then, for each polarized tweet we explored the emotion the tweet conveys. Figures 13 and 14 describe the sentiment and the emotional state of the tweets with the relative intensity of the tweets produced by Trump and Biden supporters, respectively.

What appears evident is that, on average, the tweets produced by Trump’s supporters are significantly more positive than those produced by Biden’s supporters, which devote a significant number of negative tweets to their opponent.

For what concerns the detected emotions, Trump’s supporters express *joy* and *confidence* about Trump, while *fear* about Biden’s election. Biden’s supporters, instead, show *trust* and *anticipation* in having Biden as future president of the USA, with a more marked presence of negative emotions about Trump, like *anger*, *disgust* and *sadness*.

Tables 4 and 5 show various examples of tweets including in the analysis, showing how our approach can model social media conversation from an emotional point of view.

**Table 4:** A sample of pro-Trump tweets showing different emotions

Tweet	About	Sentiment	Emotion
“First time registered voter excited to vote for@realDonaldTrump #FourMoreYears”	Trump	Positive	Joy
“#JoeBiden Democrats support domestic terrorists and exploit race and gender for political gain.I’m afraid for America #PennsylvaniansForTrump”	Biden	Negative	Fear
“You are a disgrace to politicize the death of these people, but obviously you don’t care. #JoeBiden #BidenHarris”	Biden	Negative	Disgust
“#realDonaldTrump If anyone can do it, you can. Best President ever! Godspeed sir. #AmericaFirst #MAGA2020”	Trump	Positive	Trust

**Table 5:** A sample of pro-Biden tweets showing different emotions

Tweet	About	Sentiment	Emotion
“We all need to #VoteBiden to make this happen #VOTE”	Biden	Positive	Anticipation
“#realDonaldTrump You are a racist and a loser. #TrumpIsALoser #RacistTrump”	Trump	Negative	Disgust
“Today is a sad day. News reports are talking about 200,000 Americans dead from Covid-19 so far #TrumpKnew #COVID19 ”	Trump	Negative	Sadness
“I want empathy and decency in the White House. #BidenHarris2020ToSaveAmerica”	Biden	Positive	Trust

## Conclusions and final remarks

The widespread use of social media can be exploited to extract useful information concerning people’s behaviors and interactions.

In this paper we presented an in-depth analysis of the posts published on Twitter during the 2020 US election campaign, jointly exploiting several techniques for topic discovery, opinion mining and emotion analysis in a unified analysis workflow, with the aim of outlining an accurate representation of this political event from different points of view. In particular, we extracted the main discussion topics following a clustering-based approach, monitoring their weekly impact on social media conversation. Moreover, we leveraged IOM-NN to estimate the polarization of Twitter users regarding the two main candidates Donald Trump and Joe Biden, both in real-time and by focusing on the main US swing states. We also investigated the temporal dynamics of the online discussion, combining it with the polarization information coming from IOM-NN, in order to study how users’ publishing behavior reflected external events, over time, in relation to their political orientation. Finally, we exploited sentiment analysis and text mining techniques to discover the relationship between the user polarization, determined with the aid of IOM-NN, and the sentiment expressed in referring to the different candidates, thus modeling political support of Twitter users from an emotional viewpoint.

Experimental evaluation shows that in the early weeks online conversation focused on the relationship between Trump and Covid-19 pandemic and on the nomination of Judge Amy Coney Barrett as Associate Justice of the US Supreme Court. In the following weeks, instead, the focus of the online conversation shifted to other topics including the accusations leveled to Hunter Biden and the criticism leveled against Trump linked to his position about the climate crisis and veterans. Regarding the political polarization of public opinion, IOM-NN was able to achieve meaningful estimates of the voting intentions of social media users, which makes it a valid solution to go beyond traditional opinion polls, by providing relevant insights useful to understand the dynamics of the election campaign. One major drawback of this approach lies in different possible platform biases, such as usage biases due to the distribution of users of a social media platform in terms of gender, age, culture and social status, as well as technical biases related to platform policies about data availability and restrictions imposed in some areas of the world. Finally, as for the analysis of the emotional state of social users, we found out that the tweets produced by Trump’s supporters are significantly more positive than those produced by Biden’s supporters. In particular, *i*) Trump’s supporters express *joy* and *confidence* about Trump, while *fear* about Biden’s election; *ii*) Biden’s supporters show *trust* and *anticipation* in having Biden as future president of the USA, with a more marked presence of negative emotions about Trump, like *anger*, *disgust* and *sadness*.

As future work, we will apply the presented analysis workflow to other scenarios, such as product adoption analysis and reputation evaluation of companies. In fact, it can be easily generalized to different use cases, as it is not tied to any specific application domain, and only relies on the representativeness of the analyzed posts. Moreover, we can integrate other techniques in our workflow, introducing new steps aimed at improving the quality of the achieved results. As an example, a hashtag recommendation model can be used for enriching the information content of the analyzed data, since keyword-based approaches like IOM-NN are strongly dependent on the availability of consistent hashtags in social media posts (Cantini et al. 2021).

## Data Availability

The data that support the findings of this study are publicly available. In particular, this data was gathered using Twitter APIs (https://developer.twitter.com.) and is hosted on Github (https://github.com/SCAlabUnical/USA2020).

## Footnotes

1 https://scalab.dimes.unical.it/usa-2020/ (text in Italian).

2 https://textblob.readthedocs.io/en/dev/.

3 http://sentistrength.wlv.ac.uk/.

4 https://stanfordnlp.github.io/CoreNLP/.

5 https://github.com/SCAlabUnical/IOM-NN.

6 http://www.electproject.org/2020g.

7 https://www.statista.com/statistics/265647/share-of-us-internet-users-who-use-twitter-by-age-group/.

8 https://www.statista.com/statistics/265643/share-of-us-internet-users-who-use-twitter-by-gender/.

9 https://github.com/SCAlabUnical/USA2020.

10 https://scalab.dimes.unical.it/usa-2020/ (text in Italian).

11 https://www.270towin.com/2020-polls-biden-trump/.
